# Contrasting Computational Models of Mate Preference Integration Across 45 Countries

**DOI:** 10.1038/s41598-019-52748-8

**Published:** 2019-11-15

**Authors:** Daniel Conroy-Beam, David M. Buss, Kelly Asao, Agnieszka Sorokowska, Piotr Sorokowski, Toivo Aavik, Grace Akello, Mohammad Madallh Alhabahba, Charlotte Alm, Naumana Amjad, Afifa Anjum, Chiemezie S. Atama, Derya Atamtürk Duyar, Richard Ayebare, Carlota Batres, Mons Bendixen, Aicha Bensafia, Boris Bizumic, Mahmoud Boussena, Marina Butovskaya, Seda Can, Katarzyna Cantarero, Antonin Carrier, Hakan Cetinkaya, Ilona Croy, Rosa María Cueto, Marcin Czub, Daria Dronova, Seda Dural, Izzet Duyar, Berna Ertugrul, Agustín Espinosa, Ignacio Estevan, Carla Sofia Esteves, Luxi Fang, Tomasz Frackowiak, Jorge Contreras Garduño, Karina Ugalde González, Farida Guemaz, Petra Gyuris, Mária Halamová, Iskra Herak, Marina Horvat, Ivana Hromatko, Chin-Ming Hui, Jas Laile Jaafar, Feng Jiang, Konstantinos Kafetsios, Tina Kavčič, Leif Edward Ottesen Kennair, Nicolas Kervyn, Truong Thi Khanh Ha, Imran Ahmed Khilji, Nils C. Köbis, Hoang Moc Lan, András Láng, Georgina R. Lennard, Ernesto León, Torun Lindholm, Trinh Thi Linh, Giulia Lopez, Nguyen Van Luot, Alvaro Mailhos, Zoi Manesi, Rocio Martinez, Sarah L. McKerchar, Norbert Meskó, Girishwar Misra, Conal Monaghan, Emanuel C. Mora, Alba Moya-Garófano, Bojan Musil, Jean Carlos Natividade, Agnieszka Niemczyk, George Nizharadze, Elisabeth Oberzaucher, Anna Oleszkiewicz, Mohd Sofian Omar-Fauzee, Ike E. Onyishi, Baris Özener, Ariela Francesca Pagani, Vilmante Pakalniskiene, Miriam Parise, Farid Pazhoohi, Annette Pisanski, Katarzyna Pisanski, Edna Ponciano, Camelia Popa, Pavol Prokop, Muhammad Rizwan, Mario Sainz, Svjetlana Salkičević, Ruta Sargautyte, Ivan Sarmány-Schuller, Susanne Schmehl, Shivantika Sharad, Razi Sultan Siddiqui, Franco Simonetti, Stanislava Yordanova Stoyanova, Meri Tadinac, Marco Antonio Correa Varella, Christin-Melanie Vauclair, Luis Diego Vega, Dwi Ajeng Widarini, Gyesook Yoo, Marta Zaťková, Maja Zupančič

**Affiliations:** 1 Department of Psychological and Brain Sciences, University of California, Santa Barbara, Santa Barbara, 93106 United States of America; 2 Department of Psychology, University of Texas at Austin, Austin, 78712 United States of America; 3 Institute of Psychology, University of Wroclaw, Wroclaw, 50-137 Poland; 4 Smell & Taste Clinic, Department of Otorhinolaryngology, TU Dresden, Dresden, 01307 Germany; 5 Institute of Psychology, University of Tartu, Tartu, 50090 Estonia; 6 Department of Mental Health, Faculty of Medicine, Gulu University, Gulu, Uganda; 7 English Language Department, Middle East University, Amman, 11181 Jordan; 8 Department of Psychology, Stockholm University, Stockholm, 10691 Sweden; 9 Institute of Applied Psychology, University of the Punjab, Lahore, 54590 Pakistan; 10 Department of Sociology and Anthropology, University of Nigeria, Nsukka, 410002 Nigeria; 11 Deparment of Anthropology, Istanbul University, Istanbul, 34452 Turkey; 12 North Star Alliance, Kampala, Uganda; 13 Department of Psychology, Franklin and Marshall College, Lancaster, 17603 United States of America; 14 Department of Psychology, Norwegian University of Technology and Science (NTNU), Trondheim, 7491 Norway; 15 EFORT, Department of Sociology, University of Algiers 2, Algiers, 16000 Algeria; 16 Research School of Psychology, Australian National University, Canberra, 2601 Australia; 17 EFORT, Department of Psychology and Educational Sciences, University of Algiers 2, Algiers, 16000 Algeria; 18 Institute of Ethnology and Anthropology, Russian Academy of Sciences, Moscow, 119991 Russia; 19 Russian State University for the Humanities, Moscow, 119991 Russia; 20 Department of Psychology, Izmir University of Economics, Izmir, 35300 Turkey; 21 Social Behavior Research Center, Faculty in Wroclaw, SWPS University of Social Sciences and Humanities, Wroclaw, 53-238 Poland; 22 Psychology Faculty (CECOS), Université Catholique de Louvain, Louvain-la-Neuve, 1348 Belgium; 23 Department of Psychology, Ankara University, Ankara, 6560 Turkey; 24 Department of Psychotherapy and Psychosomatic Medicine, TU Dresden, Dresden, 1069 Germany; 25 Grupo de Psicología Política y Social (GPPS), Departamento de Psicología, Pontificia Universidad Católica del Perú, Lima, 15088 Perú; 26 Deparment of Anthropology, Sivas Cumhuriyet University, Sivas, 58140 Turkey; 27 Facultad de Psicología, Universidad de la República, Motevideo, 11200 Uruguay; 28 Instituto Universitário de Lisboa (ISCTE-IUL), CIS-IUL, Lisboa, 1649-026 Portugal; 29 Department of Psychology, Chinese University of Hong Kong, Hong Kong, China; 30 Escuela Nacional de Estudios Superiores, Unidad Morelia UNAM, Morelia, 58190 Mexico; 31 Psychology Department, Universidad Latina de Costa Rica, San José, 11501 Costa Rica; 32 EFORT, Department of Psychology and Educational Sciences, University of Setif 2, Setif, 16000 Algeria; 33 Institute of Psychology, University of Pécs, Pécs, 7624 Hungary; 34 Faculty of Social Sciences and Health Care, Department of Psychological Sciences, Constantine the Philosopher University in Nitra, Nitra, 94974 Slovakia; 35 Louvain Research Institute in Management and Organisations (LOURiM), Université Catholique de Louvain, Louvain-la-Neuve, 1348 Belgium; 36 Faculty of Arts, Department of Psychology, University of Maribor, Maribor, 2000 Slovenia; 37 Department of Psychology, Faculty for Humanities and Social Sciences, University of Zagreb, Zagreb, 10000 Croatia; 38 Department of Educational Psychology and Counseling, University of Malaya, Kuala Lumpur, 50603 Malaysia; 39 Organization and Human Resource Management, Central University of Finance and Economics, Beijing, 100081 China; 40 Psychology Department, University of Crete, Rethymno, 70013 Greece; 41 Faculty of Education, University of Primorska, Koper, 6000 Slovenia; 42 VNU University of Social Sciences and Humanities, Vietnam National University, Hanoi, 100000 Vietnam; 43 Department of Psychology, IMCB, F-10/4, Islamabad, 44000 Pakistan; 44 Center for Research in Experimental Economics and Political Decision Mating, Department of Economics, University of Amsterdam, Amsterdam, 1081 Netherlands; 45 Department of Psychology, Università Cattolica del Sacro Cuore, Milan, 20123 Italy; 46 Department of Experimental & Applied Psychology, Vrije Universiteit Amsterdam, Amsterdam, 1081 Netherlands; 47 Department of Social Psychology, University of Granada, Granada, 18010 Spain; 48 Department of Psychology, University of Delhi, Delhi, 110021 India; 49 Department of Animal and Human Biology, Faculty of Biology, University of Havana, Havana, Cuba; 50 Department of Psychology, Faculty of Arts, University of Maribor, Maribor, 2000 Slovenia; 51 Department of Psychology, Pontifical Catholic University of Rio de Janeiro, Rio de Janeiro, 22451-000 Brazil; 52 Department of Social Sciences, Free Unviersity of Tbilisi, Tbilisi, 2 Georgia; 53 Faculty of Life Sciences, University of Vienna, Vienna, 1090 Austria; 54 School of Education, Universiti Utara Malaysia, Sintok, 6010 Malaysia; 55 Department of Psychology, University of Nigeria, Nsukka, 410002 Nigeria; 56 Institute of Psychology, Vilnius University, Vilnius, 1513 Lithuania; 57 Department of Psychology, University of British Columbia, Vancouver, V6T 1Z4 Canada; 58 Mammal Vocal Communication & Cognition Research Group, University of Sussex, Brighton, BN1 9RH United Kingdom; 59 Institute of Psychology, University of the State of Rio de Janeiro, Rio de Janeiro, 21941-901 Brazil; 60 Department of Psychology, Faculty for Humanities and Social Sciences, UNATC-CINETIc, Romanian Academy, Bucharest, 30167 Romania; 61 Department of Environmental Ecology, Comenius University, Bratislava, 842 15 Slovakia; 62 Institute of Zoology, Slovak Academy of Sciences, Bratislava, 845 06 Slovakia; 63 The Delve Pvt Ltd, Islamabad, 44000 Pakistan; 64 School of Psychology, University of Monterrey, San Pedro Garza Garcia, 66238 Mexico; 65 Department of Psychology, Faculty for Humanities and Social Sciences, University of Zagreb, Zagreb, 10000 Croatia; 66 Center for Social and Psychological Sciences, Institute of Experimental Psychology SAS, Bratislava, 841 04 Slovakia; 67 Department of Applied Psychology, Vivekananda College, University of Delhi, Delhi, 110021 India; 68 Department of Management Sciences, DHA Suffa University, Karachi, 75500 Pakistan; 69 School of Psychology, P. Universidad Catolica de Chile, Santiago, 8331150 Chile; 70 Department of Psychology, South-West University “Neofit Rilski”, Blagoevgrad, 2700 Bulgaria; 71 Department of Experimental Psychology, Institute of Psychology, University of São Paulo, São Paulo, 05508-030 Brazil; 72 Department of Communication, University Prof. Dr. Moestopo (Beragama), Jakarta, 10270 Indonesia; 73 Department of Child & Family Studies, Kyung Hee University, Seoul, 024-47 Republic of Korea; 74 Faculty of Social Sciences and Health Care, Department of Psychological Sciences, Constantine the Philosopher University in Nitra, Nitra, 94974 Slovakia; 75 Department of Psychology, Faculty of Arts, University of Ljubljana, Ljubljana, 1000 Slovenia

**Keywords:** Human behaviour, Sexual selection

## Abstract

Humans express a wide array of ideal mate preferences. Around the world, people desire romantic partners who are intelligent, healthy, kind, physically attractive, wealthy, and more. In order for these ideal preferences to guide the choice of actual romantic partners, human mating psychology must possess a means to integrate information across these many preference dimensions into summaries of the overall mate value of their potential mates. Here we explore the computational design of this mate preference integration process using a large sample of *n* = 14,487 people from 45 countries around the world. We combine this large cross-cultural sample with agent-based models to compare eight hypothesized models of human mating markets. Across cultures, people higher in mate value appear to experience greater power of choice on the mating market in that they set higher ideal standards, better fulfill their preferences in choice, and pair with higher mate value partners. Furthermore, we find that this cross-culturally universal pattern of mate choice is most consistent with a Euclidean model of mate preference integration.

## Introduction

Few decisions are more consequential than the choice of a romantic partner. Over a lifetime, whom we choose has wide-reaching importance for physical health, mental health, and financial wellbeing^1–4^. Over deep time, mate choice directly influences reproduction, lending sexual selection power to shape our mate selection psychologies. These facts have motivated much research on human mating, particularly on human mate preferences. From this work, we know that people around the world express preferences for potential mates who are kind, healthy, intelligent, and more^5^. Nonetheless, despite this success in documenting the content of human preferences, little work has explored how, computationally, humans integrate their multiple ideals in order to evaluate and select actual partners. To address this limitation, here we combine computer simulations with a large cross-cultural sample to examine the integration of mate preferences into evaluations of mate value. We pit alternative hypotheses against each other, comparing eight different competing models for integrating mate preferences and selecting romantic partners using agent-based models and empirical data on actual mate choices from 45 countries (*n* = 14,487). We find that one model, a Euclidean model of preference integration, consistently explains the human data better than the other seven, providing a robust model of human mate preference integration around the world.

Humans express ideal preferences for a wide array of traits in potential mates. Marlowe described Hadza hunter-gatherer mate preferences as falling into seven distinct dimensions^6^. Buss had participants across 37 countries rank a list of 18 preferences^5^. The best-validated mate preference instrument requests participants to report preferences on 38 dimensions, themselves thought to represent at least three underlying factors^7^. The breadth of human preferences makes great sense from an evolutionary perspective: for humans, a romantic partner is a potential reproduction, cooperation, and parenting partner. Throughout human evolutionary history, who an individual mated with would have had important consequences for their reproductive success. Consequently, selection would have strongly favored the evolution of adaptations that motivated humans to be discerning in mate choice.

The multiplicity of mate preferences, however, introduces a decision problem of its own. No individual is likely to ever encounter a potential mate who fulfills all of their many ideals. In mate choice, each person encounters an array of imperfect potentials, each fulfilling just a subset of their mate preferences. Selecting among these imperfect potential mates requires a psychology that can, for each potential mate, integrate information across preferences into a summary estimate of that person’s overall value as a mate.

This integration process is a critical step in the causal pathway from ideal preferences to actual relationships. But despite empirical discoveries of the content of people’s mate preferences, human mating research has generated less insight into how, computationally, human psychology integrates these multiple preferences to guide choices. The default assumption is that of linear combination, where preferences differentially weight the contributions of traits to mate value much like slopes in a regression formula^8^. Some work suggests the existence of complex curvilinear functions^9^. Another model proposes that preferences set up a series of hurdles which potential mates must successively surmount^10^. Although some of these proposed mate preference integration algorithms have been extensively modeled^11^, they have rarely been tested against one another or, crucially, against actual human data.

Prior work exploring mate preference integration in human samples has found that a Euclidean algorithm is a good model for how human mating psychology integrates preferences into estimates of mate value. This algorithm works by representing preferences and potential mates as points within a *p*-dimensional preference space, where *p* is the number of preferences to be integrated, and computing mate value as inversely proportional to the distance between these points^12^. Such Euclidean distances predict attraction to potential mates and do so better than a variety of alternative models for predicting attraction^13^. People’s actual chosen partners tend to fall short Euclidean distances from their ideal preferences, consistent with the hypothesis that they selected these mates according to Euclidean integration. Further, people high in mate value according to Euclidean calculations show evidence of experiencing greater power of choice on the mating market, as would be expected if others were estimating their mate value in a Euclidean fashion. Consistent with prior verbal models of assortative mating for mate value^14,15^, these high Euclidean mate value individuals pair with partners who better fulfill their ideal preferences, set higher ideal standards in mate choice, and tend to be paired with higher mate value partners^12^. Finally, discrepancies in Euclidean mate value within ongoing relationships predict feelings of relationship dissatisfaction^16^.

Altogether, this work suggests that a Euclidean algorithm is a good model for how human mating psychology integrates preferences in mate choice. However, thus far this research has suffered an important limitation: all prior research on Euclidean mate preference integration has been conducted on American samples. If the Euclidean algorithm reflects the operation of a psychological adaptation, created by selection to guide the choice of romantic partners, we should be able to show that this algorithm is a human universal, applicable as a good model of mate choice across cultures.

To test this hypothesis, we administered a brief mate preference questionnaire to *n* = 14,487 people across 45 countries from all inhabited continents around the world. This requested participants report their ideal preferences in a long-term romantic partner on five dimensions: kindness, intelligence, health, physical attractiveness, and financial prospects. These represent a subset of the preferences people are thought to use in guiding their mate choices; these five were chosen to represent preferences that are either universally ranked as highly important in mate choice (kindness, intelligence, and health) or universally ranked as differently important to males and females (physical attractiveness and resources)^5^. Participants additionally reported their own standing on each of these five dimensions as well as the standing of their actual long-term romantic partner if they had one.

We combine these cross-cultural data with agent-based models to compare Euclidean mate-preference integration to seven other models. Five of these model alternative preference integration algorithms, including a linear combination model to capture the most commonly assumed integration algorithm^8^; an aspiration threshold model to reflect models that propose preferences are integrated as thresholds^10,17^; a polynomial model used to model complex non-linear functions^9^; a curvilinear model which uses a sinusoidal function to similarly model non-linear functions using fewer parameters than the polynomial model; and finally, a cosine similarity model used to represent a similarity metric similar to but distinct from the Euclidean distance. In addition to these five alternative models of mate preference integration, we compared two null models in which mate preferences are disconnected from choice: one model in which agents select their mates randomly with respect to their preferences and a second model in which agents select their mates randomly and then update their preferences to match their chosen partners.

In each of these models, agents produce offspring with their chosen partners and we allow these populations to evolve over time in order to observe the consequences of mate choice according to different methods of mate preference integration. We then compare these simulated populations to our human samples to determine which model of mate choice produces the best approximation of real-world data. In this comparison, we focus on the pattern of four effects previously found to be characteristic of Euclidean mate preference integration: namely, strong mate preference fulfillment in Euclidean terms, but also a tendency for higher mate value people to achieve stronger mate preference fulfillment, set higher ideal standards, and pair with higher mate value partners^12^. We first assess whether this pattern evolves robustly in models of mate choice across parameter settings. Next, we determine whether this pattern replicates across cultures within our human samples. Finally, we assess whether this pattern is actually best explained by Euclidean mate preference integration or whether it can be similarly accounted for by alternative models. If Euclidean mate preference integration provides a good model of human mating psychology around the world, we should be able to show that Euclidean mate value affords power of choice on the mating market across cultures and does so in a way most consistent with Euclidean models of mate choice.

## Results

We first analyzed a series of evolutionary agent-based models to examine the consequences of mate choice according to Euclidean mate preference integration. Agents in these models possessed a set of 20 traits and 20 preferences corresponding to these traits, each drawn initially from random uniform distributions. Each agent computed their attraction to all opposite sex agents as inversely proportional to the Euclidean distance between their own preference vector and each agent’s trait vector. Agents then selected each other as mates based on these attractions. To accomplish this pairing, the model computed a mutual attraction matrix by computing the product of attraction values for all possible couples. The most mutually attracted couple then paired and was removed from the mating pool; this process iterated until all possible couples formed. This is a relatively simple model that is easy and fast to implement but still accomplishes mutual pairing on the basis of attraction. This was chosen over more complex models, such as the Gale-Shapley algorithm^18^, as it makes relatively few assumptions about the nature of the choice process—for instance, unlike the Gale-Shapley algorithm, this model does not assume that females are passive in mate choice.

Finally, agents reproduced with their chosen partners, producing offspring who inherited their preferences and traits. The number of offspring generated during reproduction was governed by an “energy” variable, itself calculated as inversely proportional to the summed deviation between the agent’s trait vector and a randomly generated optimal trait vector. Agents reproduced in proportion to their energy value, yielding a selection pressure in favor of optimal traits and preferences for those traits. The optimal value for each trait was drawn from a random uniform distribution, meaning that higher trait values were not invariably better and that agent populations had to evolve preferences that targeted more beneficial traits. We ran these models 50 times each under nine different parameter settings, crossing three levels of mutation rate and three levels of selection strength.

**Figure 1:**
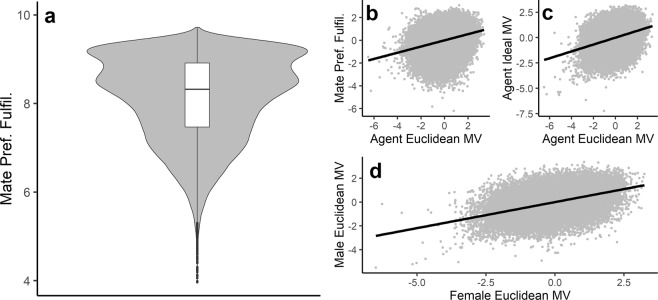
Results of agent-based models of mate choice based on Euclidean preference integration across parameter settings. In these models, agents tend to strongly fulfill their preferences in Euclidean terms (**a**); furthermore, higher mate value agents more strongly fulfill their mate preferences (**b**), set higher mate value ideal standards (**c**), and pair with higher mate value partners (**d**). All variables were standardized within model run prior to plotting for plots (**b–d**).

Figure 1 shows the results of these models across all parameter settings. A pattern of four effects characterizes the consequences of Euclidean mate preference integration. First, when agents select mates according to Euclidean mate preference integration, they tend to strongly fulfill their mate preferences in Euclidean terms. We calculate mate preference fulfillment as the Euclidean distance between an agent’s own preferences and their actual mate’s traits, transformed such that higher values indicate greater mate preference fulfillment. This is equivalent to the unique mate value of an agent’s partner to that agent^13^. In these terms, mate preference fulfillment was strong across model runs: *M* = 8.14, 95% CI [8.06, 8.21], indicating 81.4% of maximum possible preference fulfillment.

Second, three correlations indicate that agents higher in overall Euclidean mate value—that is, those that embody the preferences of the opposite sex in general^13^—experience greater power of choice on the mating market. Overall Euclidean mate value is calculated as the distance between an agent’s own trait vector and the average preference vector of the opposite sex, transformed such that higher values indicate greater mate value. Agents higher in this mate value measure tended to better fulfill their preferences in mate choice, *r*_*mean*_ = 0.27, 95% CI [0.26, 0.28]. The mate preferences of these high mate value agents also tended to target higher mate value partners. We calculated the overall Euclidean mate value of each agent’s ideal partner as the Euclidean distance between an agent’s own preference vector and the average preference vector of that agent’s same-sex competitors. This value reflects the ideal standards or “choosiness” of each agent. Higher mate value agents did indeed set higher ideal standards in that the mate value of their ideal partners was higher on average, *r*_*mean*_ = 0.34, 95% CI [0.33, 0.35]. Consistent with higher mate value agents setting higher ideal standards and more strongly fulfilling their preferences, agents tended to mate assortatively for overall mate value, *r*_*mean*_ = 0.45, 95% CI [0.44, 0.47]: higher mate value agents tended to pair with higher mate value partners.

**Figure 2:**
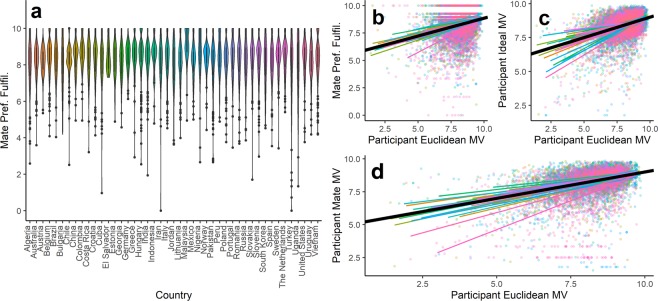
The pattern of mate choice effects from the cross-cultural human sample. Participants across cultures strongly fulfill their mate preferences across countries (**a**). Higher mate value participants furthermore more strongly fulfill their preferences (**b**), set higher mate value ideals (**c**), and tend to be paired with higher mate value partners (**d**). Colored lines represent trend lines for different countries; dots represent individual participants; the black line represents the overall trend across all countries.

Precisely the same pattern of effects emerges in each of the 45 countries in the human cross-cultural sample (Fig. 2). We calculated the same preference fulfillment and mate value measures from the agent-based models within countries of the cross-cultural sample. Overall mate preference fulfillment was high across cultures, ranging from a low in Turkey of *M* = 7.73, 95% CI [7.62, 7.84] to a high in Malaysia of *M* = 9.09, 95% CI [8.98, 9.20]. Across countries, people high in Euclidean mate value also experienced greater power of choice on the mating market. A multilevel model, with participants nested within countries and with random slopes and intercepts, shows that across all countries, people high in Euclidean mate value tended to more strongly fulfil their mate preferences, *β* = 0.26, *SE* = 0.02, *p* < 0.001. Random slopes for this effect ranged from *β* = 0.16 in Brazil to *β* = 0.49 in Turkey. Participants higher in Euclidean mate value also set higher mate value ideals across countries, *β* = 0.38, *SE* = 0.02, *p* < 0.001. Country-level random slopes for this effect ranged from *β* = 0.16 in South Korea to *β* = 0.73 in Uganda. Just as in the agent-based models, participants were mated assortatively for overall mate value, *β* = 0.35, *SE* = 0.02, *p* < 0.001. Random slopes for this effect ranged from *β* = 0.21 in Indonesia to *β* = 0.77 in Turkey.

The close correspondence between the cross-cultural sample and the agent-based models suggests that Euclidean mate preference integration is a good model of human preference integration psychology. Furthermore, this correspondence is relatively unique to models incorporating Euclidean mate preference integration. Supplementary Fig. S1 in the supplementary information compares the pattern of four effects found in the Euclidean agent-based model and cross-cultural sample to the seven alternative agent-based models. Some of these models produce comparable power of choice correlations, but not the same overall degree of mate preference fulfillment (aspiration, cosine, curvilinear, linear, polynomial); other models produce comparable degrees of overall mate preference fulfillment, but not the same power of choice correlations (preference updating).

These qualitative differences, although suggestive, are not sufficient to compare some of the more subtle quantitative differences between the agent-based models. To compare these models quantitatively to the human data, we employed a two-step, out-of-sample predictive accuracy procedure. First, within each run of each agent-based model (ABM), we trained a multilevel regression model that used agent mate value to predict (1) agent mate preference fulfillment, (2) ideal mate value, and (3) partner mate value. Second, we then used these ABM-trained regression models to predict the human data sight unseen, measuring the fit of these ABM-trained models to the human cross-cultural sample. To provide an assessment of model fit relative to best possible fit, we furthermore compared the ABM-trained regression models to a regression model both trained and tested on the human cross-cultural sample itself. Stronger fit between the human cross-cultural data and the ABM-trained regression models implies that the agent-based models are more accurately reproducing the trends present in the human data and that these models are therefore more plausible approximations of actual human mating psychology.

**Figure 3:**
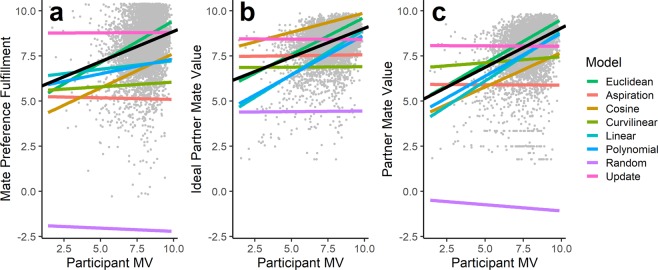
An example of the ABM-trained regression models used to compare each agent-based model to the cross-cultural human data. Models use agent mate value to predict mate preference fulfillment (**a**), ideal partner mate value (**b**), and actual partner mate value (**c**). All models come from the parameter setting in which mutation rate and selection strength were set to their lowest values. Colored lines represent predicted values from each ABM-trained regression model; the black line represents the best-fit regression line trained and tested on the cross-cultural data; gray dots represent observations from the cross-cultural data. Data points are jittered to reduce overplotting.

Figure 3 presents an example of the fit of these ABM-trained regression models. These example fits come from the parameter setting in which both mutation rate and selection strength were set to their lowest values. Among the ABM-trained regression models, the models based on the Euclidean agent-based model visually appear to best fit the human data and most closely approximate the best-fit regression line in terms of both slope and intercept, suggesting the Euclidean agent-based model is best approximating real-world human mating phenomena. To compare models quantitatively, we computed two fit indices: (1) the root mean squared error (RMSE) of the model predicted values with respect to the human data and (2) the correlation between model predicted values and observed values. We computed 95% confidence intervals for each of these values based on variation across model runs and use these confidence intervals to compare the relative fit of each of the models to the cross-cultural sample.

**Figure 4:**
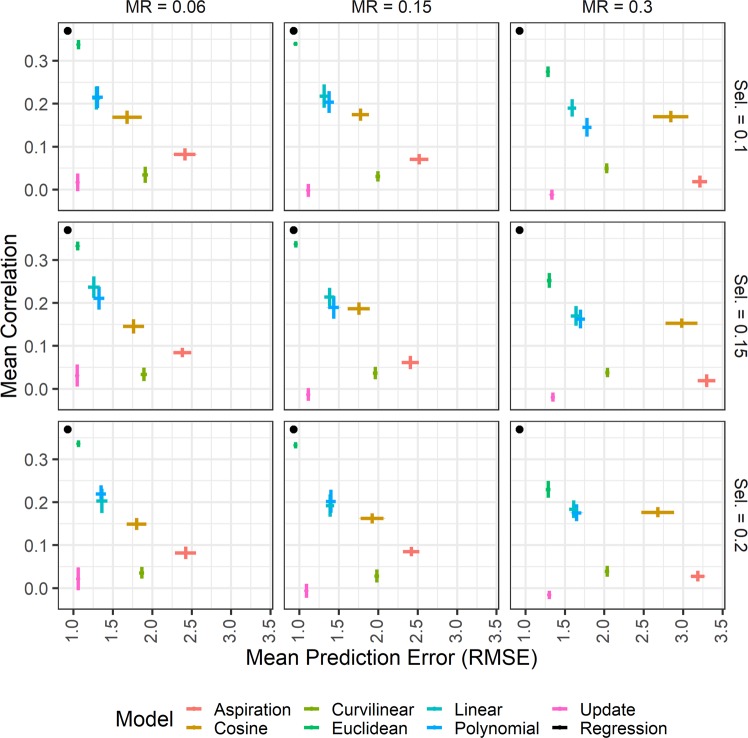
Comparing the fit indices of each ABM-trained regression model to the cross-cultural human data across models and across parameter settings. Points closest to the top left corner of each panel represent better model fit across indices. The random model had very poor fit and is therefore excluded from the plot for clarity. Error bars represent 95% confidence intervals in both directions. “MR” = mutation rate; “Sel.” = selection strength.

Figure 4 plots these model fit statistics across parameter settings; Supplementary Table S1 presents the values of all fit statistics. Consistent with Fig. 3, the regression models trained on the Euclidean agent-based models provide the best approximation of the cross-cultural human data across all parameter settings. The Euclidean-trained regression models had lower RMSE values relative to all models under all parameter settings except at the lowest mutation rates. Here, the Euclidean models did not have a significantly lower RMSE than the preference-updating models, as indicated by the completely overlapping confidence intervals. This strong correspondence between the human cross-cultural data and the preference-updating model in terms of RMSE occurs because the preference-updating model is able to produce a strong fit to the mean value of the human data. However, it does not reproduce any of the correlations between variables found in the human cross-cultural sample (Fig. 3). Accordingly, even in the best fitting models, the correlation between the preference-updating model predicted values and the human cross-cultural data is significantly lower than the correlation produced by the Euclidean agent-based models. In fact, the Euclidean agent-based model produces a predicted value correlation significantly higher than all other agent-based models in all nine parameter settings.

Overall, the Euclidean agent-based model produces the best model fit statistics of all agent-based models in nine parameter settings for one fit measure and in six parameter settings for the other. No other agent-based model ever significantly outperforms the Euclidean regression model on either model fit index. Furthermore, the Euclidean agent-based model is the only model that ever approximates the model fit statistics produced by the cross-cultural sample’s own best-fit regression line.

Further analyses indicate these results are robust even when addressing several limitations of these primary analyses. First, in six of the primary agent-based models, agents pair on the basis of the mutual attraction of both agents in each possible couple. An alternative model could treat the least attracted member of each possible couple as a limiting factor. For this reason, we ran a second set of agent-based models in which agents pair based on the attraction of the least attracted member of each possible couple (see Supplementary Note 1). These models produce identical results as the primary models, suggesting again that Euclidean mate preference integration is the most realistic model within this set.

Second, in the primary model, each agent evaluates all opposite-sex agents within the population—meaning each agent evaluates a total of 100 potential mates. This population size was chosen to be comparable to Dunbar’s predicted human social group size^19^. Nonetheless, this may be a large set of potential mates to assume each agent considers; furthermore, the assumption that all individuals in the population mutually know one another is unrealistic. For this reason, we ran a separate set of models in which each agent evaluates just a random subset of the potential mates in the local population (Supplementary Note 2). The Euclidean agent-based models continue to provide the best fit to the human data even in these models of incomplete mate search, suggesting that the results of the primary model are not an artifact of the assumption of complete mate search.

Third, all primary analyses were based exclusively on self-reported participant data. Although some of these effects are known to replicate in samples employing partner and third-party reports^20^, the self-reported nature of the cross-cultural data may have introduced important biases. To address this concern, we repeated all analyses for a subset of the sample for which we could identify actual romantic dyads, and therefore analyze self- and partner-report composites, rather than self-report alone. These return similar results, with Euclidean mate preference integration still providing the best approximation of the human cross-cultural data.

## Discussion

Human mating research has generated much understanding of the content of human preferences, but less understanding of how these multiple preferences are integrated and applied to evaluate and select real partners. Here we present the first cross-cultural exploration of human mate preference integration. We replicate and extend prior research in finding that, despite variability in a range of sociodemographic features including language, religion, climate, and political systems across cultures, human mating markets around the world share two universal features: (1) in mate choice, people strongly fulfill their mate preferences in Euclidean terms and (2) power of choice on the mating market is afforded to those highest in Euclidean mate value. Across parameter settings and across countries, this universal pattern of human mating data is most consistent with agent-based models of mate choice based on Euclidean mate preference integration. This lends support to the Euclidean preference integration algorithm as an approximation of human mate preference integration across cultures.

This research provides three key contributions to the understanding of human mating. First, it sheds light onto the computational design of human mate choice psychology. The application of ideal preferences to evaluate potential mates is a critical but poorly understood step in the process of mate choice. By finding evidence that the Euclidean algorithm provides a good model of the psychology underlying this integration around the world, this research provides insight into the design features of the adaptations that govern human mating behavior and the nature of human mating markets.

Second, this research supports the cross-culturally robust importance of mate value to power of choice on the mating market. The concept of mate value has long been a focus of abstract theorizing, where it has been assumed to be a central construct for understanding human mating behavior^21^. Consistent with this, prior studies have observed that mate value predicts, for instance, standards in mate choice^15^, the fulfillment of mate preferences^22^, and the mate value of chosen romantic partners^23^. However, this work has been conducted almost exclusively on American or Western samples. The current study provides new evidence for these long-hypothesized phenomena on a broad cross-cultural scale, lending empirical weight to the sometimes intangible construct of mate value.

Finally, this research represents a step forward in validating new research tools provided by a Euclidean model of mating psychology. As we have demonstrated, researchers can use Euclidean calculations to compute measures of mate preference fulfillment, mate value, and mate value discrepancies—each of which have been theorized to be important for regulating the formation and development of romantic relationships^24,25^. Validating the Euclidean algorithm as a good model of mating psychology across cultures contributes to validating these measures as useful tools for researchers interested in studying the nature of human mate choice and relationships.

Despite these contributions, this research has limitations that leave open directions for future research. First, we have focused so far on what is universal in mate choice across countries, but differences between the countries in our cross-cultural sample are just as important a topic for future research. Although the 45 countries in our sample appear to share something similar to Euclidean preference integration in common, they undoubtedly differ on many other aspects of mate choice including local sex ratios, typical age at marriage, or the influence of outside parties on choice. This variability likely contributes to important variability in mating outcomes; for instance, although mate preference fulfillment was high in all countries, it did vary substantially across countries as well. Discovering the sources of such variability in human mating outcomes must be a central goal of future research.

Second, here we explored an important early step in mate choice: the integration of mate preferences into estimates of mate value. However, this work still leaves open the question of precisely how people use mate value estimates to select among potential mates. We modeled this final choice process using a simplified abstraction based on mutual attraction. However, there are more complex models of the mate choice process in the prior literature^11,26–28^. These models provide detailed hypotheses concerning the nature of mate choices, but are often agnostic to the nature of mate preference integration. Future research could combine these two lines of inquiry by modifying models of the mate choice process alongside models of mate preference integration in order to determine the most plausible overall sequence of human mate selection.

Furthermore, the mating system we explored focused exclusively on long-term, monogamous pair bonds. However, humans flexibly select from a menu of mating strategies that range from the highly committed relationships we studied here to short-term, uncommitted, opportunistic affairs^29^. Additionally, although most human marriages are monogamous, most human societies have historically allowed at least some degree of polygyny^30^. Different mate preference integration algorithms may be more useful in these lower investment mating contexts. For instance, satisficing algorithms might perform better in contexts where access to a larger quantity of partners provides greater benefits than pursuing fewer, higher quality partners. Future research should explore mate preference integration across a wider range of human mating strategies to assess the applicability of alternative models across the diversity of human relationships.

Third, we studied mate preference integration only in the context of already ongoing romantic relationships. One concern with this method is that participants may deceive themselves into believing their partner is a better fit to their preferences by, for example, adjusting their preferences after-the-fact to better match their chosen partner^31^. Although our preference-updating agent-based model suggests that this process is not sufficient to fully explain our human data, a more straightforward test of hypothesized mate preference integration algorithms would come from designs that allow researchers to predict mate choices at one time point from preferences at an earlier time point. This could come from studies that apply longitudinal designs in which participants report mate preferences when they are single^31^.

Fourth, for practical reasons, we were only able to measure five dimensions of mate preferences from participants in the cross-cultural sample. However, people around the world readily express many more preferences than just these five. Furthermore, many of our models assume that all people base their choices on all of these preferences and that they weight each preference equally to the others. These are not necessary assumptions. Future research should continue to compare models of mate preference integration using broader sets of preferences and allowing for individual participants to differently omit or weight specific preference dimensions.

Relatedly, the Euclidean algorithm potentially uses a large amount of information to form evaluations of potential mates in that it can, in principle, integrate any number of preferences into a summary mate value estimate. This information-hungry model of mate selection appears to contrast with heuristic models of decision making which find that decisions in other contexts are best supported by relatively few cues^32^. However, mate selection’s proximity to reproduction means, in natural selection’s eyes, it is among the most important decisions an organism will ever make. Especially in human long-term mating, where romantic partners have a variety of avenues to influence one another’s reproduction^33^, the benefits of scrutinizing partners on more dimensions might outweigh the costs of added decision-making complexity.

Additionally, just because Euclidean integration can accommodate a large number of preferences does not mean that it does. The exact dimensionality of human preferences is an open question. Although mate preference questionnaires often include several items, it is likely that individual cues are themselves integrated into higher-order summary evaluations^10^; for example, body shape cues such as waist-to-shoulder ratio, waist-to-hip ratio, and BMI being integrated into overall bodily attractiveness estimates^34^. Several higher-order preference taxonomies exist, proposing human preferences can be organized around, for instance, two^35^, three^7^, or four^36^ major dimensions. Mapping the precise contents and structure of human ideals will be essential for constructing more thorough and accurate models of human mate choice.

Furthermore, although the Euclidean algorithm performed best among the preference integration algorithms compared here, we only compared a total set of six hypothesized algorithms. These represent a broad sampling of possibilities, including previously proposed models. Even so, there are other algorithms future research could consider which may perform even better than Euclidean integration. These could include combinations of the algorithms tested here, for instance models in which individuals winnow their pool of potential mates according to thresholds but then make final choices based on linear combinations. They could also include elaborations on these models, for instance a Euclidean model that weights preference dimensions differentially based on relative importance. Future research must continue to generate candidate preference integration algorithms and could continue to apply the out-of-sample prediction method used here to compare models in order to ultimately identify more accurate models of human mate preference integration.

Additionally, we compared all models on their ability to reproduce relationships expressed exclusively in terms of Euclidean mate value. This allowed us to test between the several alternative models on a common metric. We do observe in the human data both strong Euclidean mate preference fulfillment and evidence of greater power of choice afforded to participants higher in Euclidean mate value. Any accurate model of human mate preference integration must be able to account for these empirical observations regardless of whether that model assumes a role of Euclidean mate preference integration in mate choice. Nonetheless, a more thorough approach would compare the different models of mate preference integration according to the definitions of mate value implied by each model; for instance, comparing the predictions of the linear combination model using an analogous linear combination in the human data, rather than Euclidean mate value. This would be feasible in the human cross-cultural sample only for cosine mate preference integration, as this requires only measures of ideal preferences and corresponding traits. However, calculating cosine mate value in the cross-cultural sample yields mate values that have a strong ceiling effect and a highly restricted range: the median cosine mate value is *Mdn* = 9.96 out of 10 and 75% of participants have mate values above 9.92. This extremely restricted range would make comparing alternative models difficult. It also on its own suggests cosine preference integration is an implausible model of human mate preference integration as evaluations of potential mates do not appear similarly restricted^13^.

Comparing the other models of mate preference integration similarly would require information on the relative importance of alternative preferences to compute the mate value implied by the linear combination model; minimum and maximum preferences to compute aspiration threshold mate value; and more sophisticated preference functions to compute mate value according to the polynomial and curvilinear models. Limitations on survey length precluded us from collecting such data in the human cross-cultural sample. However, future research could collect these data from participants and compare all models of mate choice across all definitions of mate value.

In summary, we find that a Euclidean algorithm provides a good model of human mate preference integration in a large sample of participants from countries around the world. This represents an important advance in a human mating literature with a greater understanding of the nature and determinants of preferences and less understanding of their application to actual mate choice. Our study provides the largest and only cross-cultural examination of mate preference integration. In doing so, our results provide a foundation for future work in the form of a candidate model of human mate preference integration psychology. Moreover, this work provides a body of methodological tools for future research on the nature of mate choice and preference integration, including new agent-based models, model comparison tools, and methods for calculating mate preference fulfillment and mate value. Finally, this work presents the first broad, cross-cultural demonstration of the role of mate value in human mate choice. Samples from around the world show evidence of high mate value people experiencing greater power of choice on the mating market; these individuals set higher ideal standards, better fulfill their mate preferences, and disproportionately pair with high mate value partners. Altogether, this research provides new insight into the universal design of human mating psychology and human mating markets.

## Methods

### Agent-based models

We constructed and analyzed a series of agent-based models of mating markets underpinned by different mate preference integration algorithms. All models generated populations of 200 agents at the start of all model runs. Each agent possessed a set of 20 traits and between 20 and 80 preferences corresponding to these traits. Traits were drawn from random uniform distributions with initial values drawn between 1 and 7 in order to be comparable to Likert scales. Preferences were drawn from random uniform distributions with values between −10 and 10. This wider range for preferences relative to traits was chosen to be equally uninformative to all preference integration algorithms: restraining the starting values for preferences to 1 and 7 as for traits would provide an unfair advantage to the Euclidean model as its preferences would be relatively close to optimum by default. This would also unfairly disadvantage models that treat preferences as slopes as these models would not be capable of producing attraction to lower trait values at model start. The wider starting range for preferences helps guarantee all models have relatively equivalently poor starting conditions.

Agents were additionally assigned an energy value based on their traits; at the start of each model run, the model randomly selected a trait value as “optimal” for each trait dimension by drawing from a random uniform distribution for each trait dimension. Agents earned energy inversely proportional to their deviation from this optimal value on each trait; agents reproduced in proportion to their energy values, introducing a selection pressure in favor of trait values closer to optimum and preferences for these trait values. Finally, all agents were randomly assigned to be either male or female in equal proportion. After initialization, agents followed a life cycle in which they computed how attracted they were to one another, selected each other as mates based on these attractions, reproduced with their chosen partner, and then died. This life cycle repeated for 200 generations of simulated evolution. All models were run for 50 iterations per parameter setting, yielding 450 runs per model and 3,600 model runs in total.

#### Attraction

In the first phase of each life cycle, agents calculated how attracted they were to all opposite-sex agents using their traits, preferences, and preference integration algorithms. We simulated a total of six preference integration algorithms that combined traits and preferences into attraction values in different ways. Agents in the aspiration models had two preferences per trait; these preferences identified an ideal trait range for each dimension. To calculate attraction to a potential mate, aspiration agents determined how many of a potential mate’s traits fell within the agent’s ideal trait range. Cosine agents possessed one ideal preference per trait and calculated attraction as proportional to the cosine similarity between their own preference vector and each potential mate’s trait vector, where cosine similarity is the cosine of the angle formed by the agent’s preference vector, the potential mate’s trait vector, and the origin. Curvilinear agents had two preferences per trait, which acted as slopes in a sinusoidal function. These slopes manipulated the phase and frequency of a sine wave relating potential mate trait values to attraction values. Euclidean agents had one preference per trait and calculated attraction as the Euclidean distance between their own preference vector and each potential mate’s trait vector; shorter distances indicated greater attraction. Linear agents had two preferences per trait and calculated attraction as a linear combination of the potential mate’s trait values, with one preference acting as a slope and the other as an intercept for each trait. Finally, polynomial agents had four preferences per trait, three of which served as slopes and one of which served as an intercept in a cubic polynomial function calculating attraction from potential mate trait values.

#### Mate selection

The attraction phase in each model produced two matrices: a matrix that indicated how attractive each female agent found each male agent and a second matrix that indicated how attractive each male agent found each female agent. In six of the models, these two matrices were next multiplied elementwise to produce the mutual attraction matrix. Each cell in this matrix represented how mutually attracted all possible agent couples would be. The model next paired agents by identifying the most mutually attracted possible couple, pairing these agents, and removing them from the mutual attraction matrix. This process was iterated until all possible agent couples were formed.

We additionally ran two separate manipulations of the mate selection process. In the random models, agents calculated attraction as a Euclidean distance just as in the Euclidean model. However, rather than pairing based on these attraction values, agents were paired randomly. Finally, in the preference updating models, agents also calculated attraction as a Euclidean distance and also selected mates randomly with respect to these attraction values. However, after mate selection, these agents updated their preferences to be 90% closer to the traits of the mate they had already chosen.

#### Reproduction

Agents next produced offspring with their chosen mates. We employed roulette wheel selection to determine how many offspring each couple produced. To accomplish this, the model sampled with replacement from the population of agent couples; the size of this sample was equivalent to the initial population size. The probability that a given agent couple was sampled for each reproduction attempt was proportional to the couple’s pooled energy values. This probability was itself scaled by a selection strength parameter, which fixed the likelihood of sampling the highest energy couple relative to the lowest energy couple. We ran all models under three selection strength values: 0.10, 0.15, and 0.20, reflecting a 10%, 15%, and 20% reproductive advantage for the highest energy couple relative to the lowest energy couple respectively.

Agent couples produced one offspring each time they were sampled by this roulette wheel procedure. Offspring agents inherited each of their trait values randomly from either parent. We additionally added a small amount of random normal noise to trait values to simulate mutation. This mutation procedure is intended to simulate the effects of many, small impact mutations on trait values^37^. The amount of noise was controlled by a mutation rate parameter, which set the standard deviation of this random normal noise; we ran all models under three mutation rates: 0.06, 0.15, and 0.30, representing 1%, 2.5%, and 5% of the maximum trait range respectively. Levels of mutation rate were fully crossed with the selection strength parameter settings, yielding a total of nine parameter settings for all models. Offspring were randomly assigned to be either male or female.

#### Death

In each generation, all parent agents were erased after reproducing. Offspring agents then started the life cycle anew in the next generation, beginning with the attraction phase. All models were run for 200 generations of simulated evolution in total. The end result of each model was a population of *n* = 200 agents that represented the results of evolution under mate choice driven by varying mate preference integration algorithms.

### Cross-cultural sample

#### Participants

The cross-cultural sample consisted of *n* = 14,487 participants (7,961 female) from 45 countries representing all inhabited continents around the world. Participants from each study site were recruited from two sources: half of all participants were supposed to be recruited from university populations and half from community samples. Not all study sites kept records of participant source; we have source records for 45.83% of participants representing 22 out of the 45 countries. From study sites that kept records of participant source, 47.14% of participants did come from community samples. All participant data was collected in person using pen-and-paper surveys because internet samples tend to be unrepresentative, particularly in developing countries^38^.

Participants were *M* = 28.79 years old on average and ranged from 18 to 91 years old. Most participants (63.75%) were in a committed romantic relationship; among these, most participants were dating (49.26%), but others were also engaged (12.59%) or married (38.14%). The global cross-cultural study protocol was approved by the Ethics Committee of the Institute of Psychology, University of Wroclaw; many study sites were able to rely on this protocol for ethics approval. Sites that were not submitted additional ethics approvals to local authorities where necessary. A list of the institutional review boards and ethics committees that approved this study is available in the supplementary information. All methods were carried out in accordance with relevant guidelines and regulations; informed consent was obtained from all participants.

#### Measures

All participants reported their mate preferences in an ideal long-term mate, described as a committed, romantic partner, using a 5-item mate preference instrument. This instrument contained five 7-point bipolar adjective scales on which participants rated their ideal partner’s standing on five separate traits: intelligence, kindness, health, physical attractiveness, and financial prospects. Each trait was rated between two extremes, for instance, from 1 representing “very unkind” to 7 representing “very kind.” Participants additionally used the same rating scales to describe their own standing on each of these five traits and to rate their actual long-term partner, if they had one. This mate preference instrument was translated into local languages and back-translated by researchers at each study site.

### Data analysis

Data analysis proceeded in parallel stages for both the cross-cultural sample and the agent-based models.

#### Data processing

The first stage of analysis was processing the data in order to calculate mate preference fulfillment and Euclidean mate values. We first calculated mate preference fulfillment for both agents and human participants, where applicable, as the Euclidean distance between each individual’s preference vector and their actual chosen mate’s trait vector. These distances were transformed and scaled such that a value of 10 meant the preference and trait vectors were identical and a value of 0 meant the preference and trait vectors were as dissimilar as they could be. For agents whose preferences did not represent a trait value (aspiration, curvilinear, linear, and polynomial) their preference vector was calculated as either the center of the agent’s ideal trait range (for aspiration agents) or the trait value the agent found most attractive (for all other agents).

To calculate mate values, we first calculated the average preferences of all males and all females within country or within model run. We next calculated the overall Euclidean mate value of each agent or each participant as the Euclidean distance between each individual’s trait vector and the opposite sex’s average preference vector. We additionally calculated the mate value of each agent or participant’s mate as the distance between their mate’s trait vector and the average preference vector of the mate’s opposite sex. Finally, as a measure of choosiness, we calculated the mate value of each agent or participant’s ideal partner as the Euclidean distance between the individual’s own preference vector and the average preference vector of that individual’s sex. All mate value distances were scaled in the same manner as preference fulfillment such that a value of 10 indicated maximum possible mate value and a value of 0 indicated minimum possible mate value.

#### Model fitting

Next, data analysis proceeded in two steps. First, we calculated for all agent-based models and the cross-cultural sample the average degree of mate preference fulfillment and 3 correlations reflecting the degree to which high mate value individuals experienced higher power of choice on the mating market^12^. Average mate preference fulfillment was calculated within model run and within country. Power of choice on the mating market was quantified using either multilevel models predicting mate preference fulfillment, ideal mate value, and partner mate value from own mate value, with agents nested within model runs or participants nested within countries, or by calculating correlation coefficients between these variables within model runs.

To compare the similarity of the agent-based models to the cross-cultural sample, we used a model training and testing procedure. To accomplish this, we first trained multilevel models on each of the 72 model and parameter setting combinations. Within model runs, these models predicted agent preference fulfillment, ideal mate value, and partner mate value from the interaction of agent mate value and outcome variable type, with observations nested within agent or participant. This resulted in 50 regression models for each agent-based model (ABM) and 3,600 regression models overall. We then applied these models to predict the same values sight-unseen in the cross-cultural sample based on the participant’s actual mate value. We then quantified prediction accuracy with two metrics: (1) the root mean squared error (RMSE) between observed participant values and predicted values from the ABM-trained multilevel models and (2) the correlation between observed participant values and the predicted values from the ABM-trained multilevel models. This yielded two objective values that each reflected the degree to which each simulated mating market could account for human mate choice data across cultures. All data, model code, and analysis script is available on the Open Science Framework: https://osf.io/bz84c/?view_only=43711fed002e41e1876aecf1f3f0aa6e.

## Supplementary information

Supplementary Information
