# Supplementary material for: Contrasting Computational Models of Mate Preference Integration Across 45 Countries
Source: Sci Rep. 2019 Nov 15;9:16885. doi: 10.1038/s41598-019-52748-8 (PMC6858324; doi:10.1038/s41598-019-52748-8)
Supplement: Supplementary file 1 — Supplementary Information [file 41598_2019_52748_MOESM1_ESM.pdf]

Supplementary Information: Contrasting Computational Models of Mate Preference Integration Across 45 Countries

Daniel Conroy-Beam*[1], David M. Buss[2], Kelly Asao[2], Agnieszka Sorokowska[3,4], Piotr Sorokowski[3], Toivo Aavik[5], Grace Akello[6], Mohammad Madallh Alhababha[7], Charlotte Alm[8], Naumana Amjad[9], Afifa Anjum[9], Chiemezie S. Atama[10], Derya Atamtürk Duyar[11], Richard Ayebare[12], Carlota Batres[13], Mons Bendixen[14], Aicha Bensafia[15], Boris Bizumic[16], Mahmoud Boussena[17], Marina Butovskaya[18,19], Seda Can[20], Katarzyna Cantarero[21], Antonin Carrier[22], Hakan Cetinkaya[23], Ilona Croy[24], Rosa María Cueto[25], Marcin Czub[3], Daria Dronova[18], Seda Dural[20], Izzet Duyar[11], Berna Ertugrul[26], Agustín Espinosa[25], Ignacio Estevan[27], Carla Sofia Esteves[28], Luxi Fang[29], Tomasz Frackowiak[3], Jorge Contreras Garduño[30], Karina Ugalde González[31], Farida Guemaz[32], Petra Gyuris[33], Mária Halamová[34], Iskra Herak[35], Marina Horvat[36], Ivana Hromatko[37], Chin-Ming Hui[29], Jas Laile Jaafar[38], Feng Jiang[39], Konstantinos Kafetsios[40], Tina Kavčič[41], Leif Edward Ottesen Kennair[14], Nicolas Kervyn[35], Truong Thi Khanh Ha[42], Imran Ahmed Khilji[43], Nils C. Köbis[44], Hoang Moc Lan[42], András Láng[33], Georgina R. Lennard[16], Ernesto León[25], Torun Lindholm[8], Trinh Thi Linh[42], Giulia Lopez[45], Nguyen Van Luot[42], Alvaro Mailhos[27], Zoi Manesi[46], Rocio Martinez[47], Sarah L. McKerchar[16], Norbert Meskó[33], Girishwar Misra[48], Conal Monaghan[16], Emanuel C. Mora[49], Alba Moya-Garófano[47], Bojan Musil[50], Jean Carlos Natividade[51], Agnieszka Niemczyk[3], George Nizharadze[52], Elisabeth Oberzaucher[53], Anna Oleszkiewicz[3,4], Mohd Sofian Omar-Fauzee[54], Ike E. Onyishi[55], Baris Özener[11], Ariela Francesca Pagani[45], Vilmante Pakalniskiene[56], Miriam Parise[45], Farid Pazhoohi[57], Annette Pisanski[49], Katarzyna Pisanski[3,58], Edna Ponciano[59], Camelia Popa[60], Pavol Prokop[61,62], Muhammad Rizwan[63], Mario Sainz[64], Svjetlana Salkičević[65], Ruta Sargautyte[56], Ivan Sarmány-Schuller[66], Susanne Schmehl[53], Shivantika Sharad[67], Razi Sultan Siddiqui[68], Franco Simonetti[69], Stanislava Yordanova Stoyanova[70], Meri Tadinac[65], Marco Antonio Correa Varella[71], Christin-Melanie Vauclair[28], Luis Diego Vega[31], Dwi Ajeng Widarini[72], Gyesook Yoo[73], Marta Zaťková[74], Maja Zupančič[75]

[1]Department of Psychological and Brain Sciences, University of California, Santa Barbara, Santa Barbara, 93106, United States of America. [2]Department of Psychology, University of Texas at Austin, Austin, 78712, United States of America. [3]Institute of Psychology, University of Wroclaw, Wroclaw, 50-137, Poland. [4]Smell & Taste Clinic, Department of Otorhinolaryngology, TU Dresden, Dresden, 01307, Germany. [5]Institute of Psychology, University of Tartu, Tartu, 50090, Estonia. [6]Department of Mental Health, Faculty of Medicine, Gulu University, Gulu, Uganda. [7]English Language Department, Middle East University, Amman, 11181, Jordan. [8]Department of Psychology, Stockholm University, Stockholm, 10691, Sweden. [9]Institute of Applied Psychology, University of the Punjab, Lahore, 54590, Pakistan. 10Department of Sociology and Anthropology, University of Nigeria, Nsukka, 410002, Nigeria. [11]Deparment of Anthropology, Istanbul University, Istanbul, 34452, Turkey. [12]North Star Alliance, Kampala, Uganda. [13]Department of Psychology, Franklin and Marshall College, Lancaster, 17603, United States of America. [14]Department of Psychology, Norwegian University of Technology and Science (NTNU), Trondheim, 7491, Norway. [15]EFORT, Department of Sociology, University of Algiers 2, Algiers, 16000, Algeria. [16]Research School of Psychology, Australian National University, Canberra, 2601, Australia. [17]EFORT, Department of Psychology and Educational Sciences, University of Algiers 2, Algiers, 16000, Algeria. [18]Institute of Ethnology and Anthropology, Russian Academy of Sciences, Moscow, 119991, Russia. [19]Russian State University for the Humanities, Moscow, 119991, Russia. 20Department of Psychology, Izmir University of Economics, Izmir, 35300, Turkey. [21]Social Behavior Research Center, Faculty in Wroclaw, SWPS University of Social Sciences and Humanities, Wroclaw, 53-238, Poland. [22]Psychology Faculty (CECOS), Université Catholique de Louvain, Louvain-la-Neuve, 1348, Belgium. [23]Department of Psychology, Ankara University, Ankara, 6560, Turkey. [24]Department of Psychotherapy and Psychosomatic Medicine, TU Dresden, Dresden, 1069, Germany. [25]Grupo de Psicología Política y Social (GPPS), Departamento de Psicología, Pontificia Universidad Católica del Perú, Lima, 15088, Perú. [26]Deparment of Anthropology, Sivas Cumhuriyet University, Sivas, 58140, Turkey. [27]Facultad de

Psicología, Universidad de la República, Motevideo, 11200, Uruguay. [28]Instituto Universitário de Lisboa (ISCTE-IUL), CIS-IUL, Lisboa, 1649-026, Portugal. [29]Department of Psychology, Chinese University of Hong Kong, Hong Kong, China. 30Escuela Nacional de Estudios Superiores, Unidad Morelia UNAM, Morelia, 58190, Mexico. [31]Psychology Department, Universidad Latina de Costa Rica, San José, 11501, Costa Rica. [32]EFORT, Department of Psychology and Educational Sciences, University of Setif 2, Setif, 16000, Algeria. [33]Institute of Psychology, University of Pécs, Pécs, 7624, Hungary. [34]Faculty of Social Sciences and Health Care, Department of Psychological Sciences, Constantine the Philosopher University in Nitra, Nitra, 94974, Slovakia. [35]Louvain Research Institute in Management and Organisations (LOURiM), Université Catholique de Louvain, Louvain-la-Neuve, 1348, Belgium. [36]Faculty of Arts, Department of Psychology, University of Maribor, Maribor, 2000, Slovenia. [37]Department of Psychology, Faculty for Humanities and Social Sciences, University of Zagreb, Zagreb, 10000, Croatia. [38]Dept of Educational Psychology and Counseling, University of Malaya, Kuala Lumpur, 50603, Malaysia. [39]Organization and Human Resource Management, Central University of Finance and Economics, Beijing, 100081, China. 40Psychology Department, University of Crete, Rethymno, 70013, Greece. [41]Faculty of Education, University of Primorska, Koper, 6000, Slovenia. [42]Department of Psychology, University of Social Sciences and Humanities, Hanoi, 100000, Vietnam. [43]Department of Psychology, IMCB, F-10/4, Islamabad, 44000, Pakistan. [44]Center for Research in Experimental Economics and Political Decision Mating, Department of Economics, University of Amsterdam, Amsterdam, 1081, Netherlands. [45]Department of Psychology, Università Cattolica del Sacro Cuore, Milan, 20123, Italy. [46]Department of Experimental & Applied Psychology, Vrije Universiteit Amsterdam, Amsterdam, 1081, Netherlands. [47]Department of Social Psychology, University of Granada, Granada, 18010, Spain. [48]Department of Psychology, University of Delhi, Delhi, 110021, India. [49]Department of Animal and Human Biology, Faculty of Biology, University of Havana, Havana, Cuba. [50]Department of Psychology, Faculty of Arts, University of Maribor, Maribor, 2000, Slovenia. [51]Department of Psychology, Pontifical Catholic University of Rio de Janeiro, Rio de Janeiro, 22451-000, Brazil. [52]Department of Social Sciences, Free Unviersity of Tbilisi, Tbilisi, 2, Georgia. [53]Faculty of Life Sciences, University of Vienna, Vienna, 1090, Austria. [54]School of Education, Universiti Utara Malaysia, Sintok, 6010, Malaysia. [55]Department of Psychology, University of Nigeria, Nsukka, 410002, Nigeria. [56]Institute of Psychology, Vilnius University, Vilnius, 1513, Lithuania. [57]Department of Psychology, University of British Columbia, Vancouver, V6T 1Z4, Canada. [58]Mammal Vocal Communication & Cognition Research Group, University of Sussex, Brighton, BN1 9RH, United Kingdom. [59]Institute of Psychology, University of the State of Rio de Janeiro, Rio de Janeiro, 21941-901, Brazil. 60Department of Psychology, Faculty for Humanities and Social Sciences, UNATC-CINETIc, Romanian Academy, Bucharest, 30167, Romania. [61]Department of Environmental Ecology, Comenius University, Bratislava, 842 15, Slovakia. [62]Institute of Zoology, Slovak Academy of Sciences, Bratislava, 845 06, Slovakia. [63]The Delve Pvt Ltd, Islamabad, 44000, Pakistan. [64]School of Psychology, University of Monterrey, San Pedro Garza Garcia, 66238, Mexico. [65]Department of Psychology, Faculty for Humanities and Social Sciences, University of Zagreb, Zagreb, 10000, Croatia. [66]Center for Social and Psychological Sciences, Institute of Experimental Psychology SAS, Bratislava, 841 04, Slovakia. [67]Department of Applied Psychology, Vivekananda College, University of Delhi, Delhi, 110021, India. [68]Department of Management Sciences, DHA Suffa University, Karachi, 75500, Pakistan. [69]School of Psychology, P. Universidad Catolica de Chile, Santiago, 8331150, Chile. 70Department of Psychology, South-West University "Neofit Rilski", Blagoevgrad, 2700, Bulgaria. [71]Department of Experimental Psychology, Institute of Psychology, University of São Paulo, São Paulo, 05508-030, Brazil. [72]Department of Communication, University Prof. Dr. Moestopo (Beragama), Jakarta, 10270, Indonesia. [73]Dept. of Child & Family Studies, Kyung Hee University, Seoul, 024-47, Republic of Korea. [74]Faculty of Social Sciences and Health Care, Department of Psychological Sciences, Constantine the Philosopher University in Nitra, Nitra, 94974, Slovakia. [75]Department of Psychology, Faculty of Arts, University of Ljubljana, Ljubljana, 1000, Slovenia.

*Corresponding author: Daniel Conroy-Beam, Department of Psychological and Brain Sciences, University of California, Santa Barbara, 93106; telephone: 805-893-2121; email: dconroybeam@ucsb.edu

Supplementary Note 1: Agent-Based Models Using an Alternative Mating Market Structure

Agents in six of the primary agent-based models paired based on a mutual attraction model of mate choice. In these models, the attraction matrices for each sex were multiplied together elementwise and pairing began with the most mutually attracted pair. This market structure produced strong correspondence between the agent-based models and the human data. However, to test the robustness of these results, we also ran a separate set of models with a different mating market structure.

In this alternative model, agents paired based on minimum, rather than mutual attraction. In these models, agents computed their attraction to all opposite-sex agents using their preference integration algorithms just as in the primary models. However, the model then identified the least attracted member of each possible couple rather than the mutual attraction of all possible couples. The model next paired the agents with the highest minimum in-pair attraction value, iterating this pairing until all possible couples were formed. These minimum attraction models were identical to the mutual attraction models in all other respects.

We compared the results of the minimum attraction models to the human cross-cultural sample using the same model training and testing procedure as in the primary agent-based models. Supplementary Fig. S2 shows the results of this model comparison process. Just as in the primary models, the model in which agents integrate their preferences according to a Euclidean algorithm provides the strongest fit to the cross-cultural human sample among the six alternative models of mate preference integration. The results of the primary agent-based models, in which the Euclidean algorithm produces the best approximation of the cross-cultural human data, are therefore not limited to the mutual attraction model of mate choice.

Supplementary Note 2: Agent-Based Models Incorporating Incomplete Mate Search

Agents in each of the primary models conduct a complete search of their local mating market: each agent has information on and ultimately selects from the total set of 100 potential mates that exist in their population. While this number is within estimated limits on human social group sizes, this simplified population structure constitutes a potentially unrealistic assumption for at least three reasons. First, this implicitly supposes that all individuals in the population have perfectly overlapping social networks. Second, this population structure assumes that there is no randomness within and no limitations on the mate search process. Third, this is a large set of potential mates to consider, which may be computationally implausible. To assess whether the results reported in the primary agent-based models are dependent on this assumption, we created an alternative model in which mate search is incomplete.

These incomplete search models are identical to the primary agent-based models except for just one change. When the models compute the mutual attraction matrix, a random subset of 50 potential mates are eliminated immediately for each agent. These agent couples are therefore incapable of pairing regardless of what their attraction values would have been otherwise. This elimination simulates an incomplete and partially random search of the mating market by each agent and makes it such that different agents have slightly different—albeit overlapping—social networks in that each agent functionally “knows” just a random subset of the total population.

We compared the populations produced by these incomplete search models to the human cross-cultural sample using the same training and testing procedure as used for the primary agent-based models. Supplementary Fig. S3 presents these results. Simulating incomplete search does not substantially change the relative model fits. Across all parameter settings, the Euclidean agent-based models still provide the best overall fit to the human cross-cultural sample relative to

all of the other agent-based models of mate preference integration and still provide a fit relatively similar to the regression model both trained and tested on the data itself. The relatively good performance of this model in explaining the human cross-cultural data therefore does not appear to emerge because of the assumption of complete mate search in the primary agent-based models.

Supplementary Note 3: Comparing Agent-Based Models and Human Data Beyond Self-Report

The primary agent-based models showed a strong correspondence between the Euclidean model and the human cross-cultural data. However, a limitation of the human sample is that all data is self-report: participants reported both their own traits and preferences as well as the traits of their partners, if applicable. It is possible that this led to biased reports of mates, yielding biased results.

We addressed this problem in two ways. First, the preference-updating model allowed us to test the hypothesis that biased reports of preferences (or biased perception of partners) would spuriously produce the pattern of results observed in the human cross-cultural data. Although this biased report model can produce comparable levels of mate preference fulfillment as observed across cultures, it cannot produce the correlations between participant mate value and mate preference fulfillment, ideal mate value, or partner mate value (Supplementary Figure S1; Figure 3). This suggests that rating bias alone cannot account for the correspondence between the Euclidean agent-based model and the human cross-cultural sample.

Second and furthermore, we were able to leverage the design of this study to extract partner ratings for a subset of the sample, allowing us to conduct the same tests on data that did not rely exclusively on self-report. Although data collection in the cross-cultural sample was

entirely self-report, and although participants were not specifically recruited in dyads, in some cases participants did complete the study along with their actual romantic partner. These dyadic participations were not recorded; however, we can, through participant responses, infer which participants were members of dyads rather than participating alone. We used two sets of criteria for inferring dyads from the cross-cultural human sample: a “strict” criterion and a “less-strict” criterion. For the strict criterion, we classified two participants as belonging to a dyad if they had complimentary answers on the following questions: city of residence, own age and partner age, relationship length, relationship status, whether they saw their partner in the last week, whether they met their partner in the last week, number of children, and age of youngest child. This dyad inference process additionally only searched for heterosexual couples. The less strict criterion excluded information about number and age of children under the assumption that some mated individuals could have different responses to these questions if they had children from prior relationships.

Pairing participants into dyads based on the strict criteria produced a sample of n = 394 participants belonging to 197 inferred romantic dyads. Using the less-strict criteria resulted in n = 498 participants belonging to 249 dyads. To assess the risk of pairing participants into false dyads by chance, we ran the same dyad inference procedure on a sample in which we first randomly scrambled the responses used to pair participants into dyads within city. On this scrambled data, both dyad inference procedures produced zero inferred dyads.

With dyads, we can compare agent-based models to the human samples using responses beyond self-report. Rather than relying on self-reports for self and partner traits, we calculated composite trait scores for all participants by averaging self- and partner-reports. We then conducted the same analyses on these samples as in the full cross-cultural human sample.

Supplementary Figs. S4 and S5 show the results of these analyses for the strict and less-strict dyads respectively. In both cases, most models produced substantially lower predicted-observed value correlations than the Euclidean model; the only exceptions were the linear and polynomial models. For the less-strict dyads, the linear and polynomial models produced observed-predicted value correlations comparable to or higher than the Euclidean model in most parameter settings. For the strict dyads, the linear and polynomial models produced observed-predicted value correlations that approximated the Euclidean models in only one parameter setting. However, the Euclidean model produced a lower RMSE than the linear and polynomial models across all parameter settings; it was only matched by the preference-updating model on RMSE in 3 out of the 9 parameter settings. Overall, across all 9 parameter settings and across the two model fit estimates, the Euclidean agent-based model still produced the best fit to the human cross-cultural data in both the strict and less strict dyad inference criteria. This suggests the strong correspondence between the Euclidean agent-based model and the cross-cultural human data is not an artifact of self-report data collection.

Supplementary Note 4: Institutional Review Boards and Ethics Committees that Approved this Study

Ethical Committee of the Institute of Psychology, University of Wroclaw

The Survey and Behavioural Research Ethics Committee at the Chinese University of Hong Kong

The ANU Human Research Ethics Committee at The Australian National University

The Ethical Review Board of Vrije Universiteit Amsterdam

Ethics committee of the Department of Psychology, Faculty of Humanities and Social Sciences, University of Zagreb

University of Crete Psychology Department Research Ethics Committee

South-West University Neofit Rilski, Department of Psychology

Research Ethics Committee of the University of Tartu (UTREC)

Ethical Committee of the Technical University of Dresden

Ethical Commission in Research of the ENES, UNAM, Morelia

Ethics Review Board of CUFE Business School

Scientific Council of the Institute of Ethnology and Anthropology, RAS, Moscow, Russia

Ethics Council of the University of Setif 2, Algeria

Institutional Review Board of the University of Texas at Austin

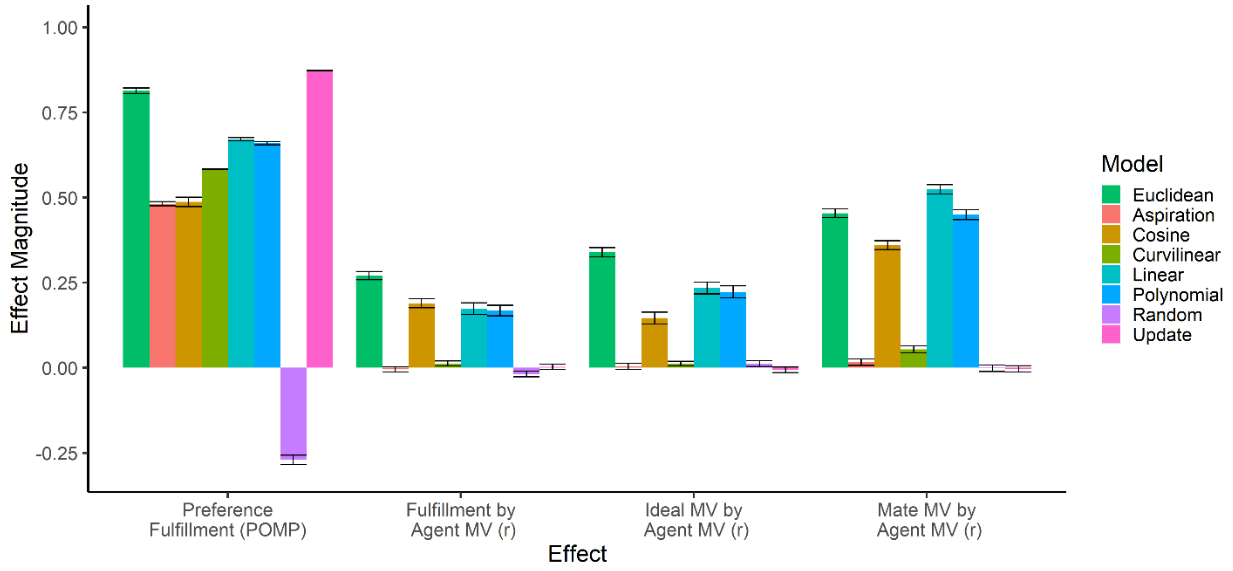

Supplementary Figure S1. A comparison of mate choice effects across all agent-based models and across parameter settings. “POMP” = percentage of maximum possible; “*r*” = correlation. Error bars represent 95% confidence intervals.

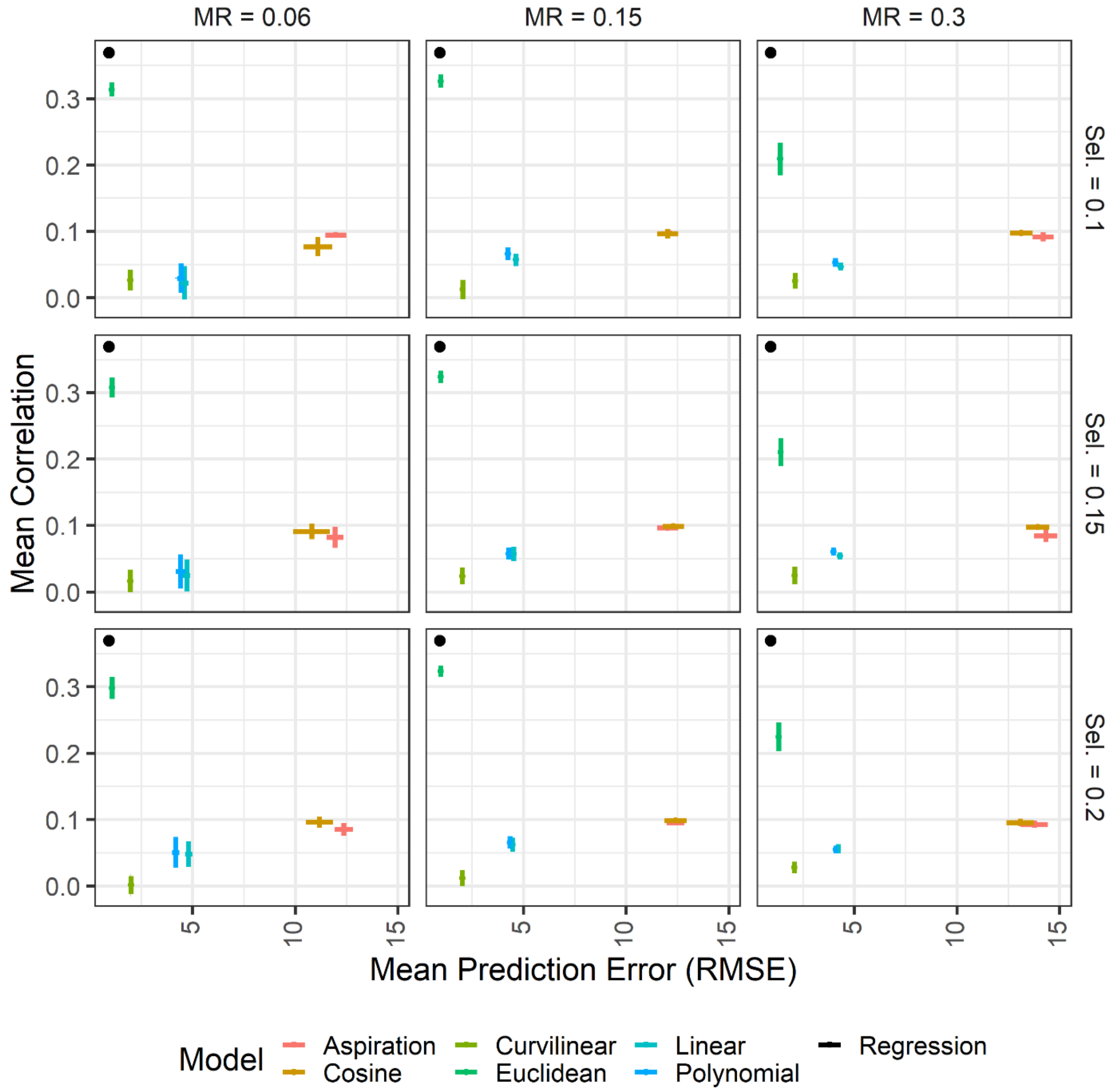

Supplementary Figure S2. Comparing the fit indices of each agent-based model to the cross-cultural human data for models in which mate choice was based on minimum attraction. Error bars represent 95% confidence intervals in both directions. “MR” = mutation rate; “Sel.” = selection strength.

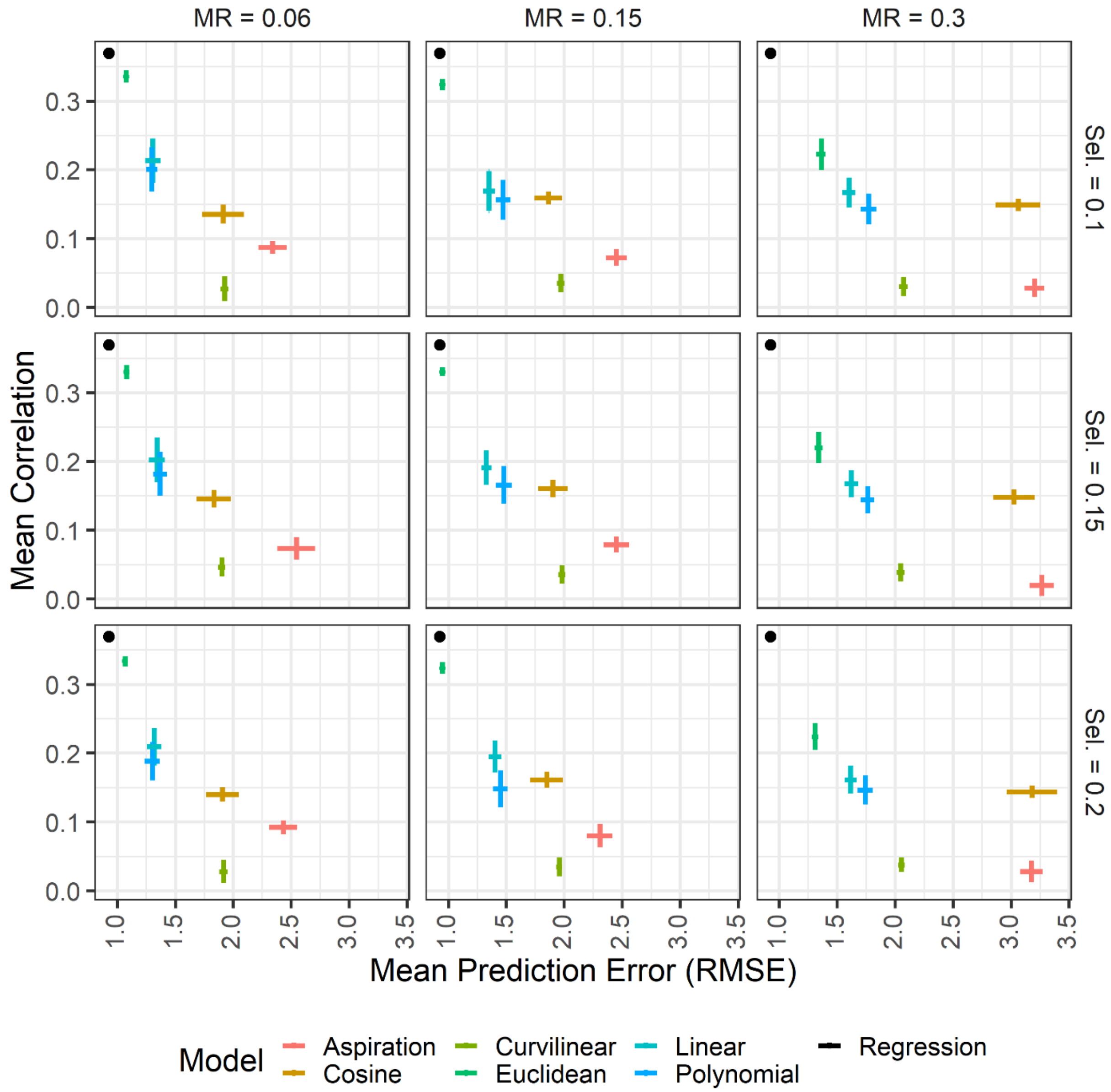

Supplementary Figure S3. Comparing the fit indices of each agent-based model to the cross-cultural human data for models in which agents conduct an incomplete search of the mating market. Error bars represent 95% confidence intervals in both directions. “MR” = mutation rate; “Sel.” = selection strength.

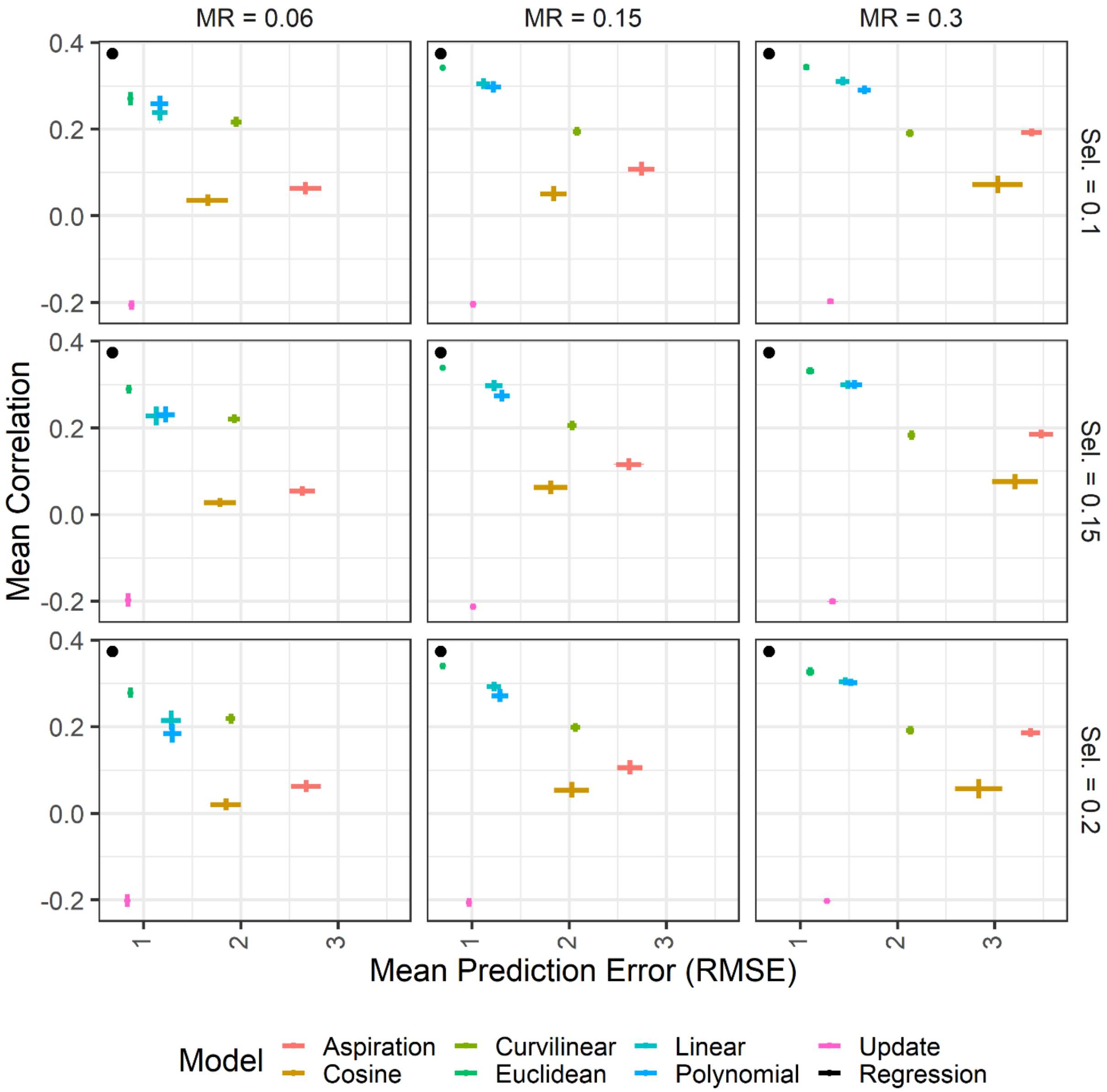

Supplementary Figure S4. Comparing the fit indices of each agent-based model to inferred dyads from the cross-cultural human data using the strict dyad inference procedure. Error bars represent 95% confidence intervals in both directions. “MR” = mutation rate; “Sel.” = selection strength.

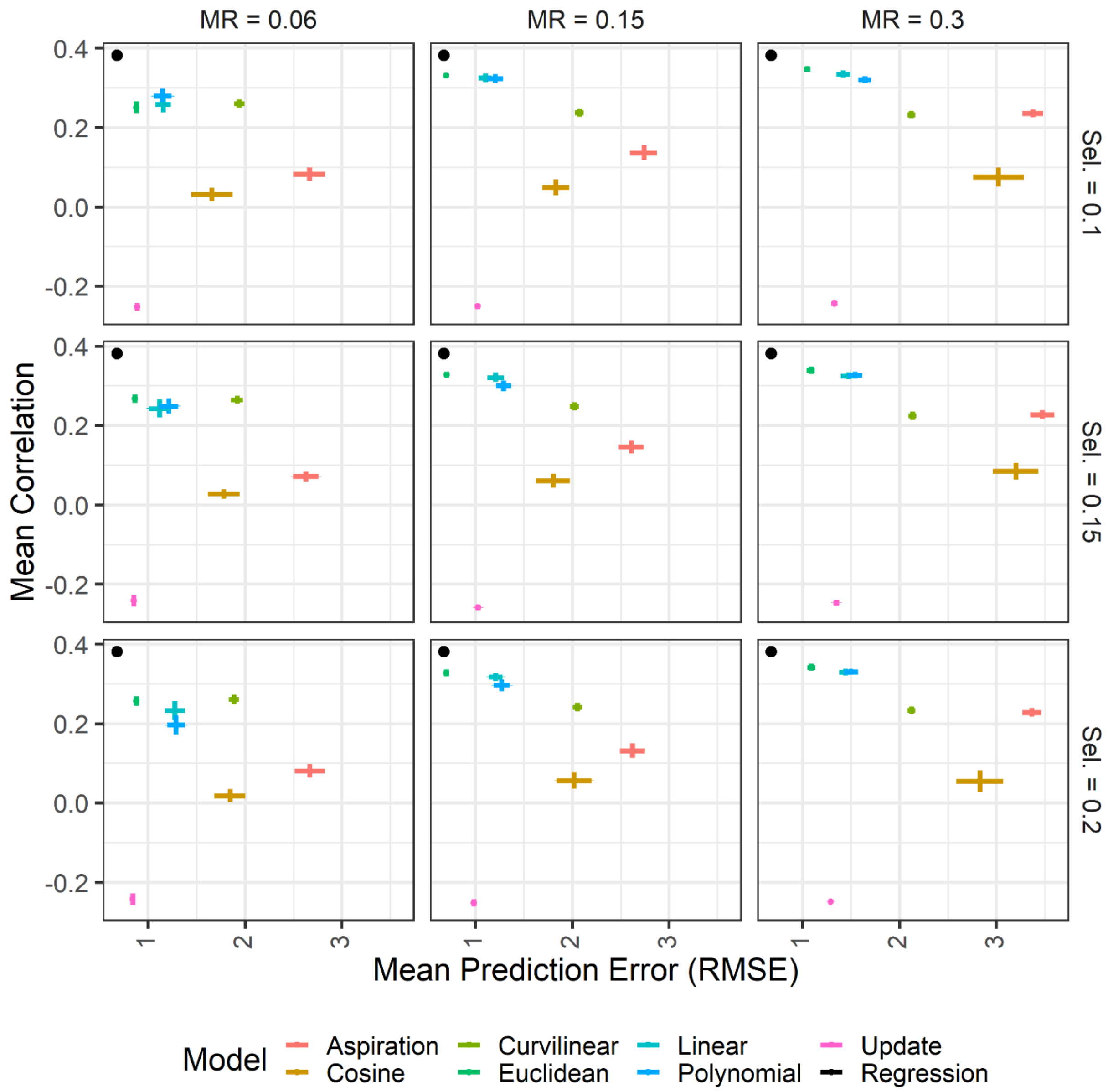

Supplementary Figure S5. Comparing the fit indices of each agent-based model to inferred dyads from the cross-cultural human data using the less-strict dyad inference procedure. Error bars represent 95% confidence intervals in both directions. “MR” = mutation rate; “Sel.” = selection strength.

Supplementary Table S1

Model fit statistics from out-of-sample prediction accuracy procedure across models and across parameter settings.

Model	Mutation Rate	Selection Strength	RMSE Mean	RMSE 95% CI	*r* Mean	*r* 95% CI
Euclidean	0.06	0.1	1.06	1.04 - 1.08	0.34	0.33 - 0.35
Euclidean	0.06	0.15	1.05	1.04 - 1.07	0.33	0.32 - 0.34
Euclidean	0.06	0.2	1.06	1.05 - 1.08	0.34	0.33 - 0.34
Euclidean	0.15	0.1	0.95	0.94 - 0.95	0.34	0.33 - 0.34
Euclidean	0.15	0.15	0.95	0.95 - 0.96	0.34	0.33 - 0.34
Euclidean	0.15	0.2	0.95	0.95 - 0.95	0.33	0.33 - 0.34
Euclidean	0.3	0.1	1.29	1.26 - 1.31	0.27	0.26 - 0.29
Euclidean	0.3	0.15	1.30	1.27 - 1.33	0.25	0.24 - 0.27
Euclidean	0.3	0.2	1.29	1.26 - 1.32	0.23	0.21 - 0.25
Aspiration	0.06	0.1	2.41	2.27 - 2.55	0.08	0.07 - 0.1
Aspiration	0.06	0.15	2.38	2.27 - 2.49	0.08	0.07 - 0.1
Aspiration	0.06	0.2	2.42	2.29 - 2.55	0.08	0.07 - 0.1
Aspiration	0.15	0.1	2.52	2.4 - 2.64	0.07	0.06 - 0.08
Aspiration	0.15	0.15	2.41	2.3 - 2.51	0.06	0.05 - 0.08
Aspiration	0.15	0.2	2.42	2.31 - 2.52	0.09	0.07 - 0.1
Aspiration	0.3	0.1	3.21	3.12 - 3.3	0.02	0 - 0.03
Aspiration	0.3	0.15	3.30	3.18 - 3.41	0.02	0 - 0.03
Aspiration	0.3	0.2	3.19	3.1 - 3.27	0.03	0.02 - 0.04
Cosine	0.06	0.1	1.68	1.49 - 1.86	0.17	0.15 - 0.18
Cosine	0.06	0.15	1.76	1.63 - 1.9	0.15	0.13 - 0.16
Cosine	0.06	0.2	1.80	1.68 - 1.93	0.15	0.14 - 0.16
Cosine	0.15	0.1	1.77	1.67 - 1.88	0.17	0.16 - 0.19
Cosine	0.15	0.15	1.75	1.61 - 1.89	0.19	0.17 - 0.2
Cosine	0.15	0.2	1.92	1.77 - 2.07	0.16	0.15 - 0.17
Cosine	0.3	0.1	2.84	2.62 - 3.06	0.17	0.16 - 0.18
Cosine	0.3	0.15	2.98	2.78 - 3.18	0.15	0.14 - 0.16
Cosine	0.3	0.2	2.68	2.47 - 2.89	0.18	0.16 - 0.19
Curvilinear	0.06	0.1	1.91	1.87 - 1.94	0.03	0.02 - 0.05
Curvilinear	0.06	0.15	1.89	1.85 - 1.93	0.03	0.02 - 0.05
Curvilinear	0.06	0.2	1.86	1.83 - 1.9	0.04	0.02 - 0.05
Curvilinear	0.15	0.1	1.99	1.97 - 2.02	0.03	0.02 - 0.04
Curvilinear	0.15	0.15	1.96	1.93 - 1.99	0.04	0.02 - 0.05
Curvilinear	0.15	0.2	1.98	1.95 - 2.01	0.03	0.01 - 0.04
Curvilinear	0.3	0.1	2.03	2 - 2.06	0.05	0.04 - 0.06
Curvilinear	0.3	0.15	2.04	2.01 - 2.06	0.04	0.03 - 0.05

Curvilinear	0.3	0.2	2.03	2.01 - 2.06	0.04	0.03 - 0.05
Linear	0.06	0.1	1.29	1.24 - 1.34	0.21	0.19 - 0.24
Linear	0.06	0.15	1.26	1.19 - 1.33	0.24	0.21 - 0.26
Linear	0.06	0.2	1.36	1.29 - 1.43	0.20	0.17 - 0.23
Linear	0.15	0.1	1.31	1.26 - 1.37	0.22	0.19 - 0.24
Linear	0.15	0.15	1.38	1.31 - 1.44	0.21	0.19 - 0.24
Linear	0.15	0.2	1.39	1.33 - 1.44	0.19	0.17 - 0.22
Linear	0.3	0.1	1.59	1.54 - 1.64	0.19	0.17 - 0.21
Linear	0.3	0.15	1.64	1.57 - 1.7	0.17	0.15 - 0.19
Linear	0.3	0.2	1.61	1.55 - 1.66	0.18	0.16 - 0.2
Polynomial	0.06	0.1	1.30	1.24 - 1.37	0.22	0.19 - 0.24
Polynomial	0.06	0.15	1.32	1.25 - 1.39	0.21	0.18 - 0.24
Polynomial	0.06	0.2	1.35	1.28 - 1.41	0.22	0.2 - 0.24
Polynomial	0.15	0.1	1.37	1.31 - 1.44	0.20	0.18 - 0.23
Polynomial	0.15	0.15	1.43	1.37 - 1.5	0.19	0.16 - 0.22
Polynomial	0.15	0.2	1.40	1.33 - 1.46	0.20	0.18 - 0.23
Polynomial	0.3	0.1	1.78	1.72 - 1.84	0.15	0.12 - 0.17
Polynomial	0.3	0.15	1.70	1.63 - 1.76	0.16	0.14 - 0.18
Polynomial	0.3	0.2	1.65	1.59 - 1.71	0.18	0.16 - 0.2
Random	0.06	0.1	8.07	7.67 - 8.47	0.08	0.07 - 0.09
Random	0.06	0.15	8.04	7.54 - 8.53	0.09	0.08 - 0.1
Random	0.06	0.2	8.62	8.04 - 9.2	0.09	0.07 - 0.1
Random	0.15	0.1	8.50	8.07 - 8.92	0.09	0.08 - 0.09
Random	0.15	0.15	8.33	8.01 - 8.65	0.08	0.07 - 0.09
Random	0.15	0.2	8.30	7.85 - 8.75	0.08	0.06 - 0.09
Random	0.3	0.1	9.07	8.65 - 9.49	0.09	0.08 - 0.09
Random	0.3	0.15	9.93	9.51 - 10.34	0.07	0.06 - 0.08
Random	0.3	0.2	9.38	8.97 - 9.79	0.08	0.07 - 0.09
Update	0.06	0.1	1.05	1.04 - 1.06	0.02	0 - 0.04
Update	0.06	0.15	1.05	1.04 - 1.06	0.03	0.01 - 0.06
Update	0.06	0.2	1.06	1.05 - 1.07	0.02	-0.01 - 0.05
Update	0.15	0.1	1.11	1.09 - 1.13	0.00	-0.02 - 0.01
Update	0.15	0.15	1.11	1.09 - 1.13	-0.01	-0.03 - 0
Update	0.15	0.2	1.09	1.08 - 1.1	-0.01	-0.02 - 0.01
Update	0.3	0.1	1.33	1.31 - 1.36	-0.01	-0.02 - 0
Update	0.3	0.15	1.35	1.32 - 1.37	-0.02	-0.03 - -0.01
Update	0.3	0.2	1.30	1.28 - 1.32	-0.02	-0.03 - -0.01
